# Electrochemical Impedance Spectroscopy Study of Ceria- and Zirconia-Based Solid Electrolytes for Application Purposes in Fuel Cells and Gas Sensors

**DOI:** 10.3390/ma17215224

**Published:** 2024-10-26

**Authors:** Małgorzata Dziubaniuk, Robert Piech, Beata Paczosa-Bator

**Affiliations:** AGH University of Krakow, Faculty of Material Science and Ceramics, Department of Analytical Chemistry and Biochemistry, Al. Mickiewicza 30, 30-059 Krakow, Poland; rpiech@agh.edu.pl

**Keywords:** gadolinium-doped ceria, scandium-doped zirconia, solid oxide electrolyte, electrochemical impedance spectroscopy, electrochemical gas sensors

## Abstract

In this study, the structural and electrochemical properties of commercial powders of the nominal compositions Ce_0.8_Gd_0.2_O_1.9_, Sc_0.1_Ce_0.01_Zr_0.89_O_1.95_, and Sc_0.09_Yb_0.01_Zr_0.9_O_1.95_ were investigated. The materials are prospective candidates to be used in electrochemical devices, i.e., gas sensors and fuel cells. Based on a comparison of the EIS spectra in different atmospheres (synthetic air, 3000 ppm NH_3_ in argon, 10% H_2_ in argon), the reactions on the three-phase boundaries were proposed, as well as the conduction mechanisms of the electrolytes were described. The Ce_0.8_Gd_0.2_O_1.9_ material is a mixed ionic–electronic conductor, which makes it suitable for anode material in fuel cells. Moreover, it exhibits an apparent and reversible response for ammonia, indicating the possibility of usage as an NH_3_ gas-sensing element. In zirconia-based materials, electrical conduction is realized by oxygen ion carriers. Among them, the most promising from an applicative point of view seems to be Sc_0.09_Yb_0.01_Zr_0.9_O_1.95_, showing a high, reversible reaction with hydrogen.

## 1. Introduction

### 1.1. The Aim of the Research

Solid electrolytes are of great interest due to their practical uses as ionic conductive components in high-temperature electrochemical devices. Examples of devices containing elements based on solid electrolytes of fluorite structure are solid oxide fuel cells (SOFCs).

So far, many ceramics based on zirconia and ceria, differing in methods and conditions of preparation as well as doping, have been tested for their electrical properties in a wide range of temperatures in an atmosphere with controlled composition. Much less common are reports on electrochemical properties, which are based on measurements of changes in electrical properties under the influence of a changing atmosphere. The results analysis provides a basis for developing hypotheses about the reactions occurring in the material, as well as to determine the optimal operating conditions as elements of electrochemical devices.

The aim of this study was to compare the structural and electrochemical properties of three sintered electrolyte materials based on ceria and zirconia available commercially, paying special attention to their potential applications in novel electrochemical devices. For the first time, using the method of electrochemical impedance spectroscopy (EIS) based on electrical response and its reversibility, hypothetical models of processes occurring in sintered specimens with gaseous ammonia and hydrogen at elevated temperatures were proposed.

### 1.2. Literature Review

One of the first ceramic materials that was used as an electrolyte in SOFC was yttria-stabilized zirconia (YSZ). Zirconia (ZrO_2_) crystallizes in the monoclinic system in normal conditions. In such a state, the material exhibits poor mechanical stability and low electrical conductivity. At temperatures above 1150 °C, ZrO_2_ undergoes a phase transition to a tetragonal crystallographic system, and above 2370 °C, to a cubic phase [1]. The transition to cubic or tetragonal phase leads to significant modification of zirconia-applicative properties. Firstly, the material is extremely durable and chemically resistant. Secondly, it shows ionic electrical conductivity realized by oxygen ion carriers [2,3]. However, operating devices constructed with elements based on ZrO_2_ at extremely elevated temperatures would not be possible.

The solution to this problem is a suitable modification of the material by decreasing the phase transition temperature. In consequence, the high-temperature crystallographic phase of the compound is stable at lower temperatures. The most popular method to achieve this aim is the addition of a precisely adjusted amount of divalent or trivalent metal atoms. The following compounds have been reported as ZrO_2_ dopants: Al_2_O_3_ [4,5,6], CaO [7,8], MgO [9,10], Y_2_O_3_ [11,12], Sc_2_O_3_ [13], Yb_2_O_3_ [14], Si_3_N_4_ [15,16,17], AlN [18,19], and CuO [20]. Such modification has a direct effect on the considerable rise of the specific electrical conductivity. This phenomenon is explained by additional oxygen vacancies creation. The Zr^4+^ ions are partially substituted by ions of lower valence, and the oxygen vacancies are formed to preserve the charge neutrality of the system. The mentioned processes can be described by the formulas written for doping with divalent and trivalent cations descendant from oxide dopant, respectively:

(1) $$\mathrm{MeO}\overset{{\mathrm{ZrO}}_{2}}{\to }{\mathrm{Me}}_{\mathrm{Zr}}^{\prime \prime }+V_{O}^{••}+O_{O}^{x},$$

(2) $${\mathrm{Me}}_{2}O_{3}\overset{{\mathrm{ZrO}}_{2}}{\to }{2\mathrm{Me}}_{\mathrm{Zr}}^{\prime \prime }+V_{O}^{••}+{3O}_{O}^{x},$$

Doping with tetravalent ions from MnO_2_ or SiO_2_ is also possible and improves the mechanical stability of the materials [21,22,23,24,25].

Zirconia-based ceramics found several practical uses. Its mechanical and chemical properties make it valuable as a prosthetic material for medicine or constructive material for industry. Moreover, high ionic electrical conductivity and temperature shock tolerance of the stabilized ZrO_2_ make it suitable for application in oxygen sensors [26,27,28,29,30], solid oxide fuel cells [31,32,33,34], ceramic components, and as catalyst or catalyst promoters in the synthesis of alcohols by hydrogenation of CO [35,36,37,38].

Scandia-stabilized zirconia is a solid electrolyte with potential application in intermediate-temperature solid oxide fuel cells (IT-SOFC) [39,40]. The main drawback of this solid electrolyte is the complex nature of phase composition according to the phase diagram [41,42]. The highest value of ionic conductivity was obtained for the addition of 9 mol% Sc_2_O_3_ [43] along with a high rate of thermal degradation, due to the formation of the tetragonal structure in the cubic matrix. The solution to this problem lies in additional material doping of small amounts of ceria (1 mol%), resulting in the stabilization of the material cubic structure. A number of studies in the ternary or even quadrupole system have been carried out so far. The most prospective of them are as follow: Zr-Ce-In [44], Zr-Zn-Ce [45], Ce-Zr-Sm [46], Zr-Cu-Co [47], Mo-Ce-Zr, W-Ce-Zr [48], Y-Zr-Ce [49,50], Cu-Zr-Ti [51], Ce-Zr-Yb [52], Sc-Ce-Zr [53,54,55], Ni-Sc-Zr [56], Sc-Zr-Yb [57], and Sc-Ce-Y-Zr [58].

The operating temperatures of fuel cells containing electrolytes based on zirconia are in the range of 900–1000 °C. Although the electrochemical processes occurring in the devices run with satisfactory efficiency, the need to use such high temperatures makes it unprofitable to generate electricity. One of the problems is the rapid degradation of cell components in extreme working conditions. Therefore, research on alternative electrolytes has been of constant interest. Among the many tested oxide ceramics with fluorite structure, the highest conductivity is shown by bismuth oxide, bismuth oxide doped with yttrium, and ceria doped with gadolinium in the amount of 20% at. For example, at 1000 °C, the reported Ce_0.8_Gd_0.2_O_1.9_ conductivity is Ω∙cm^−1^, which is one order of magnitude higher than that of YSZ electrolyte [59,60].

One of the CeO_2_ disadvantages is the tendency of cerium ion reduction, which results in an increase in the electronic conductivity component. Moreover, the material shows poor mechanical properties in the working conditions of large differences in oxygen chemical potentials. From the thermodynamic point of view, it appears that the introduction of Re_2_O_3_ admixture counteracts the reduction of Ce^4+^ ions to the Ce^3+^ form. Additionally, by introducing ions in the + III oxidation state to the cationic sub-network, oxygen vacancies are generated, contributing to conductivity improvement.

During sintering of CeO_2_, ceramic elements usually contaminate with silica (SiO_2_) originating from the quartz elements of the furnace proceeds, which is practically unavoidable. It results in increased resistance of grain boundaries regions. The contamination may also result in the segregation of impurities at the electrode–electrolyte connection. Both phenomena cause an undesirable decrease in the conductivity of the system. The addition of Gd^3+^ ions to the CeO_2_ network primarily affects grain boundary electrical properties [61,62]. In the case of samples containing small amounts of SiO_2_, higher conductivity of grain boundaries was obtained for the composition Ce_0.8_Gd_0.2_O_2-*δ*_ (CGO20) compared to Ce_0.9_Gd_0.1_O_2−*δ*_ (CGO10). On the other hand, CGO10 is characterized by a higher stability in reducing atmospheres compared to CGO20 at temperatures below 730 °C. The improvement of CGO20 stability under such conditions can be obtained by codoping with small amounts of praseodymium [63].

The influence of the type of admixture on the ceria sinterability can be predicted based on the so-called “Vegard’s Slope” [64]:

(3) $$X=\left(0.0220r_{i}+0.00015z_{i}\right),$$

where *r*_i_ is the difference between the ionic radii of the dopant metal and Ce^4+^ for the coordination number equal to 8, and *z*_i_ is the charge difference between the introduced ion and Ce^4+^ ions. The maximum amount of modifier ions that can be incorporated in network positions is inversely proportional to the *X*-factor [65,66,67,68,69,70].

In order to determine the possibility of using ceramics as a component of a fuel cell, it is necessary to describe its electrochemical properties. One of the parameters important from the applicative point of view is material conductivity. Literature reports on the electrical conductivity of ceria-based materials vary significantly due to the significant impact of preparation conditions on the final properties of sintered specimens [66,67,68,69,70,71,72,73,74]. An exemplary comparison of results for four cerium oxide-based materials at temperatures 500, 600, and 700 °C is shown in Table 1 [75]. At 500 °C, the CGO10 material has the highest conductivity value, while the yttrium-dotted sample shows the highest conductivity at temperatures of 600 °C and 700 °C.

The aim of this study was to compare the structural and electrochemical properties of three sintered electrolyte materials available commercially, paying special attention to their potential applications in novel electrochemical devices.

## 2. Materials and Methods

### 2.1. Materials Used

The investigations were conducted on commercially available powders provided by Terio [76]. The declared compositions of the investigated materials are Ce_0.8_Gd_0.2_O_1.9_ (labeled as GDC), Sc_0.1_Ce_0.01_Zr_0.89_O_1.91_ (labeled as ScCeSZ), and Sc_0.09_Yb_0.01_Zr_0.9_O_1.95_ (labeled as ScYbSZ).

The silver paste used for EIS measurements was provided by Pelco (Fresno, CA, USA).

### 2.2. Samples Preparation

**Figure 1:**
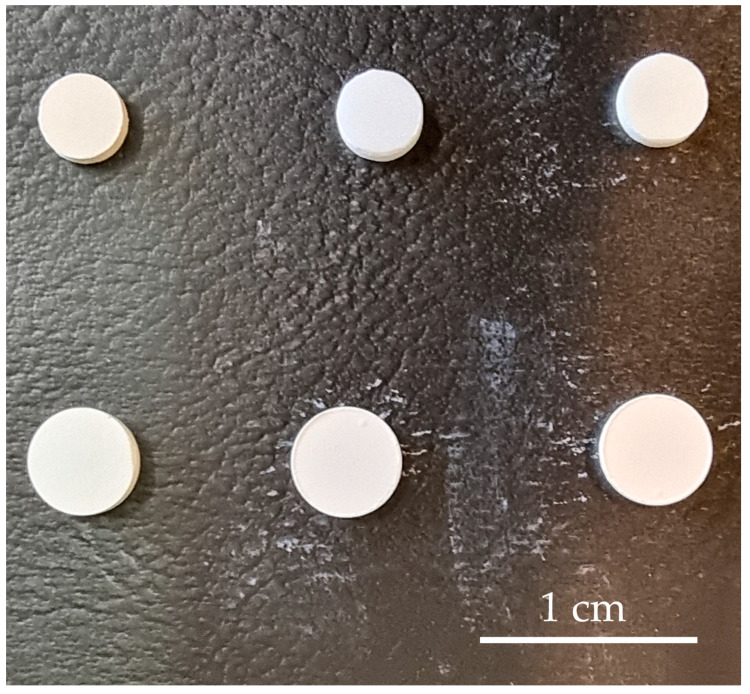
The comparison of green bodies (bottom row) with sintered specimens (upper row) in order: GDC, ScCeSZ, and ScYbSZ.

The powders were uniaxially pressed into pellets of 5 mm diameter and 0.2 g weight under 300 MPa and sintered according to the program recommended by the producer in an ambient atmosphere. In case of ScCeSZ and ScYbSZ, the sintering steps were as follows: heating from 20 °C to 300 °C for 4 h, holding at 300 °C for 1 h, heating from 300 °C to 500 °C for 2 h, heating from 1500 °C to 1660 °C for 3 h 15 min, holding at 1660 °C for 1 h, cooling from 1660 °C to 1100 °C for 2 h 30 min, and cooling from 1100 °C to 20 °C for 8 h 20 min. In the case of 20GDC, the sintering steps were as follows: heating from 20 °C to 300 °C for 3 h 20 min, holding at 300 °C for 2 h, heating from 300 °C to 1500 °C for 15 h, holding at 1500 °C for 2 h, and cooling from 1500 °C to 20 °C for 6 h. The noticeable shrinkage of specimens during the sintering process is exhibited in Figure 1.

The pellets were prepared for electrochemical impedance spectroscopy (EIS) measurements by covering both sides with Ag paste firing at 700 °C for 5 min.

### 2.3. Characterisation of Sintered Discs

The sintered specimens’ densities were measured by the Archimedes method.

Sintered pellets were examined by the X-ray diffraction (XRD) method. The measurements were performed in air at room temperature using CuK_α_ radiation (Philips X’Pert) within the 2*Θ* range of 10–90° with a scan ratio of 0.008°/s.

The surface morphology of sintered materials was observed using an ultrahigh-resolution scanning electron microscope (SEM) with field emission (FEG-Schottky emitter; Nova Nano-SEM 200, FEI Europe BV, Eindhoven, The Netherlands) cooperating with energy-dispersive spectroscopy and an X-ray analyzer (EDAX EDS, Mahwah, NJ, USA).

### 2.4. Electrochemical Property Examination by Electrochemical Impedance Spectroscopy (EIS)

**Figure 2:**
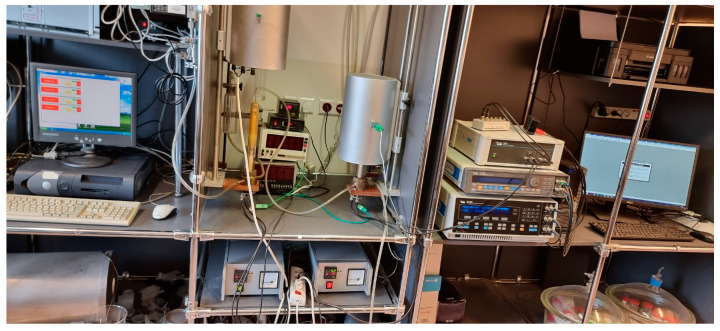
Experimental set for EIS measurements.

**Figure 3:**
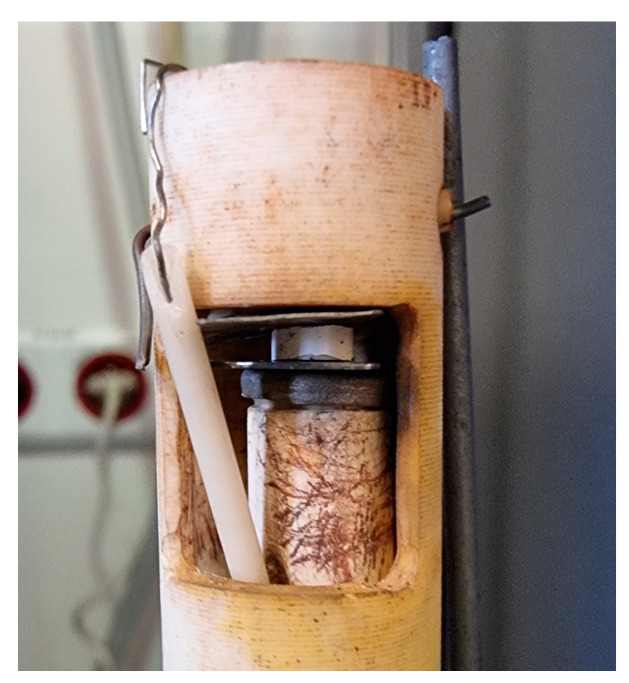
The specimen holder for EIS measurements.

The EIS measurements were performed in controlled atmospheres using a frequency analyzer (Solartron model FRA 1260, Bognor Regis, UK) coupled with a Dielectric Interface (model 1296) in a temperature range of 200–600 °C. The experimental set is presented in Figure 2 and the specimen holder in Figure 3. The frequency range was 0.1 Hz–32 MHz, and the amplitude of the sinusoidal voltage signal was 20 mV. The measurements were performed in the following atmospheres: synthetic air (series no 1, 3, and 5), 3000 ppm NH_3_ in argon (series no 2), and 10% H_2_ in argon (series no 4). Series no 3 and 5 in the synthetic air were conducted in order to determine the reversibility of the material impedance response in H_2_- and NH_3_-rich atmospheres. The values of the resistances were derived using Zview software (version 2.2, Scribner Associates, Inc., Southern Pines, NC, USA).

## 3. Results

### 3.1. Morphological Features of the Sintered Samples

GDC, ScYbSZ, and ScCeSZ densities measured by the Archimedes method were, respectively, 7.13 g/cm^3^ (~92% of theoretical density), 5.84 g/cm^3^ (~91% of theoretical density), and 5.99 g/cm^3^ (~95% of theoretical density).

**Figure 4:**
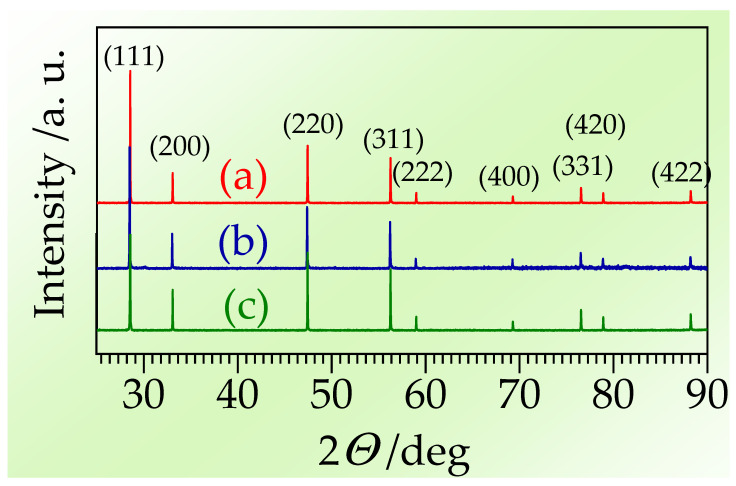
The XRD patterns with labeled peaks characteristic for cubic phase recorded for sintered samples (a) GDC, (b) ScCeSZ, (c) ScYbSZ.

Spectra from XRD are presented in Figure 4. The GDC and ScYbSZ are single-phase and crystalize in the cubic system, while the ScCeSZ specimen is composed mainly of the ZrO_2_ phase with CeO_2_ traces. The positions and intensities of the detected peaks are consistent with reference spectra [77,78].

**Figure 5:**
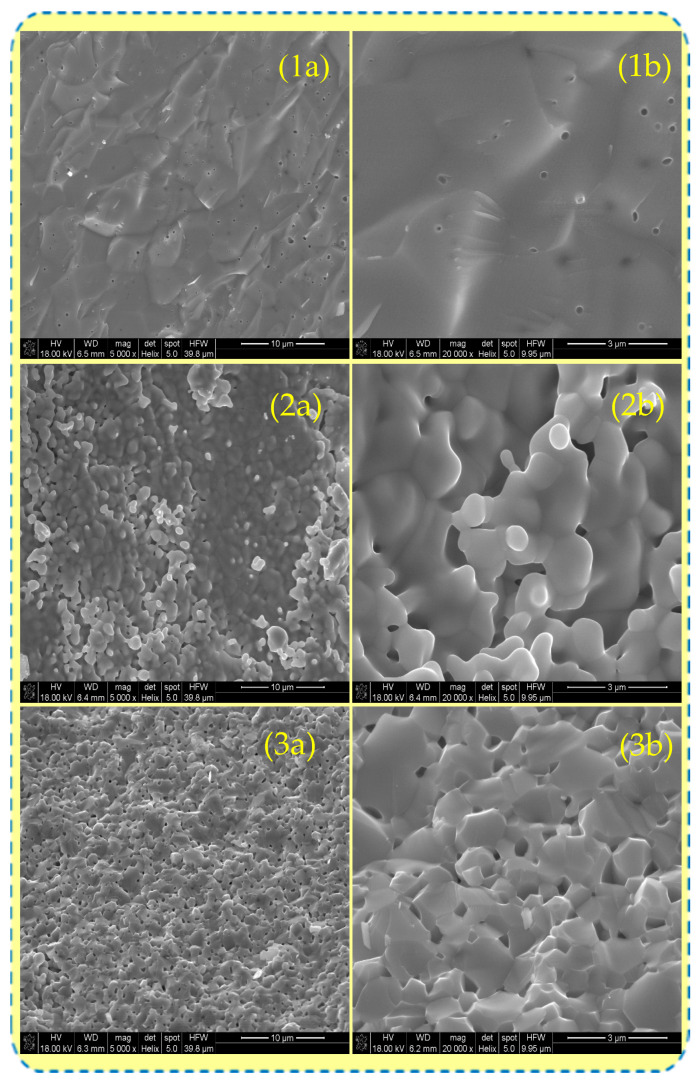
Surface morphology of sintered samples presented on SEM images at different magnifications for (**1a**,**1b**) GDC, (**2a**,**2b**) ScCeSZ, and (**3a**,**3b**) ScYbSZ.

The exemplary images of the specimens’ microstructure studied by the SEM technique are presented in Figure 5. The GDC surface is smooth almost without cracks. In this case, a few round small randomly situated pores are revealed. The images indicate high gas tightness and density of the material. ScCeSZ and ScYbSZ ceramics are composed of partly grown, melted grains characterized by ovoid edges and sizes from the range of 1–3 μm. Between grains, small pores are visible. The specific area of ScCeSZ and ScYbSZ is significantly higher than in the case of GDC.

Based on XRD spectra, the lattice parameters were calculated. The measurement of elemental compositions was performed by an EDS analyzer. The calculated data from EDS and XRD are presented in Table 2. The lattice parameter of GDC is notably higher than for ScCeSZ and ScYbSZ, which remains in accordance with theoretical predictions. The elemental analysis shows a significant deficiency in oxygen in every specimen; however, it may be due to the fact that the measurement uncertainty tends to be higher for lighter elements. Surprisingly, specimen ScCeSZ contains measurable amounts of yttrium which was not declared in the nominal composition of the material. Moreover, the amounts of scandium and cerium are much higher than those from the theoretical calculations. In the case of ScYbSZ, the determined amount of ytterbium considerably exceeds the expected value.

### 3.2. Electrochemical Properties of the Sintered Samples

**Figure 6:**
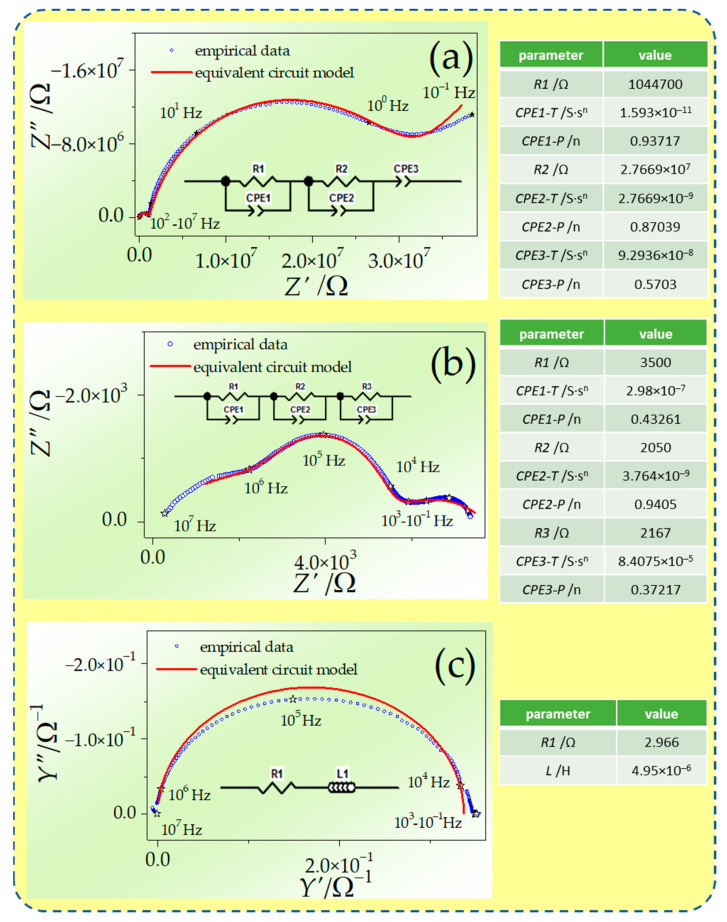
Evolution of EIS spectrum of GDC in H_2_-containing atmosphere with rising temperature; red line obtained using presented equivalent circuits for parameters values collated in tables at temperatures (**a**) 200 °C, (**b**) 500 °C, and (**c**) 550 °C.

The electrochemical properties were investigated by EIS spectra analysis realized by dedicated software. The exemplary evolution of Nyquist spectra with temperature for GDC obtained during the 4th series in the H_2_-rich atmosphere is presented in Figure 6. The figure contains the results of experimental data analysis by fitting equivalent circuits method. At a temperature of 200 °C, the spectrum is comprised of two separated semicircles in high and medium frequencies and a short fragment arc in low frequencies. The investigated material can be classified as polycrystalline ceramic. In such cases, the highest-frequency part of the spectrum corresponds with bulk properties, and the medium-frequency fragment is attributed to grain boundary behavior. The shape of the spectra indicates that the grain boundary regions are notably more resistive than bulk regions. At 500 °C, the metallic electrode becomes reversible to hydrogen. Such behavior is deduced from the semicircle shape of the spectrum fragment in low frequencies. At 600 °C, the conductivity of the specimen becomes as high as in metallic conductors. Thus, the semicircle shape is visible in the admittance plane and equivalent circuit, simulating the behavior of the specimen containing the inductor.

**Figure 7:**
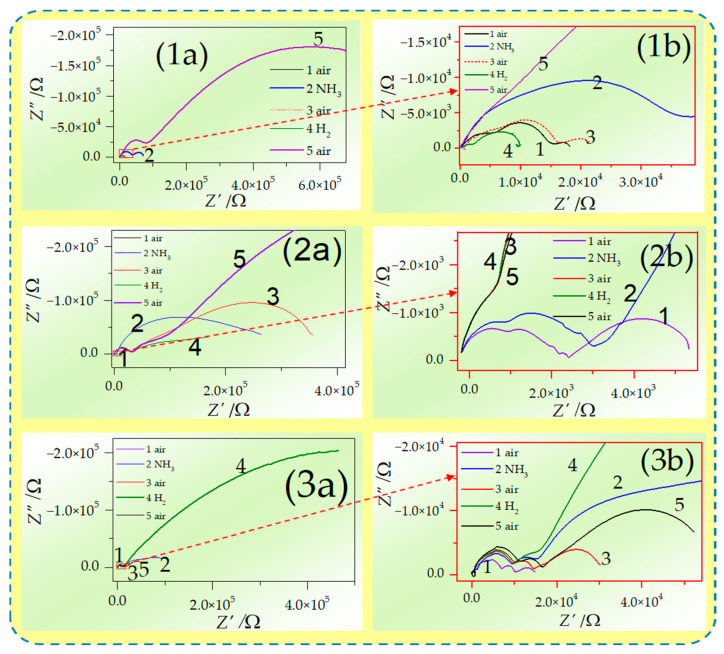
Changes in electrical properties caused by exposition on ammonia- and hydrogen-containing atmospheres presented as Nyquist spectra from EIS at 450 °C for (**1**) GDC, (**2**) ScCeSZ, and (**3**) ScYbSZ; column (**b**) contains magnified high-frequency bottom-right corner region of spectra from (**a**) column.

The exemplary sets of data obtained at 450 °C in different atmospheres for specimens in the Nyquist plane are presented in Figure 7.

**Figure 8:**
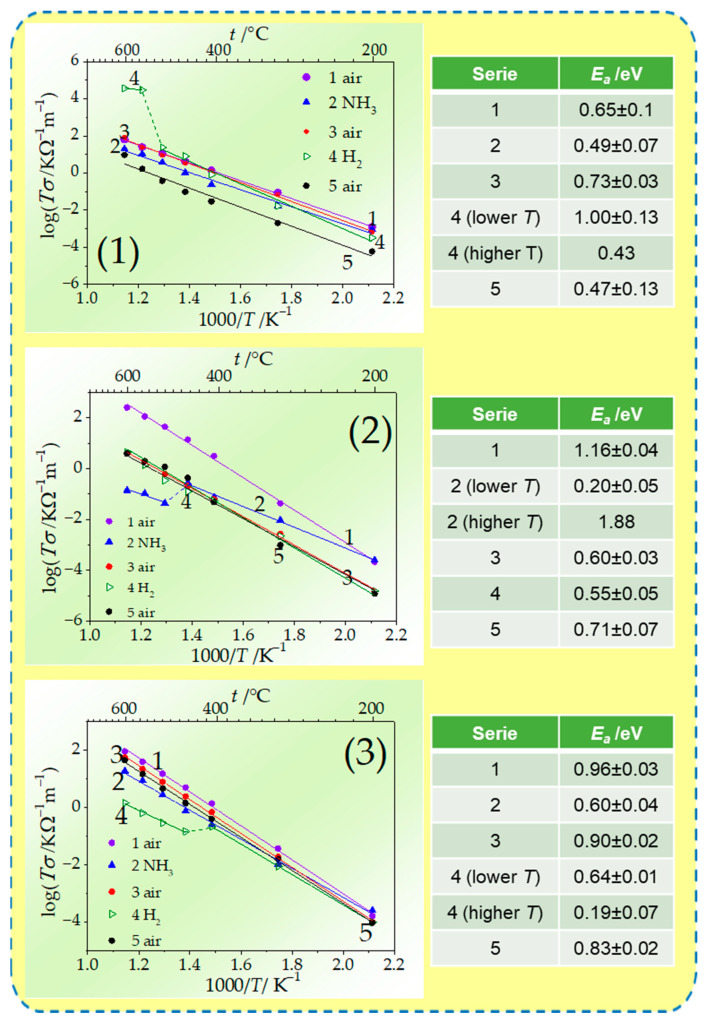
Summary of electrochemical response and reversibility of the probes for exposition on ammonia- and hydrogen-containing atmospheres presented in Arrhenius plots with calculated energy of activation values for (**1**) GDC, (**2**) ScCeSZ, and (**3**) ScYbSZ.

The specific conductivities were calculated using values of specimens’ resistance obtained by analysis of the spectra taking into account the pellet geometry. The collation of specific conductivity values obtained during the 1st series in the air, which can be considered as reference measurements of electrical conductivity before further modification of the materials, is presented in Table 3. The results are presented in Figure 8 as experimental points in Arrhenius coordinates along with lines derived from linear regression fitting. At 200 °C and 300 °C, the specimen GDC shows the highest electrical conductivity. The determined conductivities are somehow lower compared with literature data at particular temperatures, presented in Table 1. The discrepancy may arise from different sintering conditions or different initial microstructure of the materials [74]. At 400 °C, the conductivities for all the investigated samples are comparable. The best-conducting properties at higher temperatures are demonstrated by the ScCeSZ specimen, which is consistent with the literature reports [79].

The noticeable kink in the slope in the Arrhenius plots at around 550 °C for GDC and ScCeSZ and 450 °C for ScYbSZ is denoted. It indicated an order–disorder transition of oxygen vacancies, which was previously observed in doped ceria specimens [80]. However, to confirm this explanation, more sophisticated measurements by use of a high-temperature X-ray diffraction (HT-XRD) and Raman and dielectrics spectroscopy techniques in a controlled atmosphere are required. 

Additionally, based on slopes of fitted Arrhenius plots, the activation energy values for total specific conductivities were determined and presented in Figure 8. The values of 0.5 eV indicate the extrinsic character of the conductivity, while values of 1 eV indicate the intrinsic character of the conductivity.

## 4. Discussion

In a typical solid oxide electrolyte, conductivity is proportional to the relative concentration of oxygen ions, which are charge carriers in the cryllographic lattice. The conductivity is described by the equation

(4) σ=qcμ,

where

*q*—valency of mobile ions,

*c*—relative concentration of mobile ions to the number of possible positions in the lattice,

and *μ*—mobility of the ions.

In the atmosphere rich in oxygen, the reversible oxidation and reduction occur on a three-phase boundary (gas–metallic electrode–solid electrolyte) [81]:

(5) $$O_{2}+4e^{-}⇄{2O}^{2-}.$$

The above reaction is the basis for the operation of semiconductor oxygen sensors. A relationship between the oxygen partial pressure $P_{O}$ and the electrical conductivity σ of an oxide sensor can be represented by [82]

(6) $$\sigma =P_{O}^{m}A\exp(-\frac{E_{A}}{kT}),$$

where *σ* is the electronic conductivity, *A* is a constant, *E_A_* is the activation energy for conduction, and *m* is a parameter determined by both the type of the carrier (n or p) and the defects (e.g., oxygen vacancy) in the semiconductor. The additional ionic carriers appear in the lattice, increasing measured conductivity. The opposite effect of decreasing conductivity is observed in neural or reduced atmospheres due to the reversibility of the reaction (5). Taking into account what was stated above, the behavior of ScYbSZ shows the predictable and reversible response of the typical oxygen conductor to changes in oxygen partial pressure in the atmosphere.

In the case of GDC in an NH_3_-rich atmosphere, the reversible decrease in conductivity is measured, while in an H_2_-containing atmosphere, the conductivity drop is observed followed by its further decrease during the 5th series in synthetic air in a temperature range of 200–500 °C. The deterioration of conducting properties of the oxide electrolytes is predictable in the conditions of atmospheres lacking in oxygen, as it was demonstrated during the 2nd series. The results obtained in an atmosphere containing hydrogen exhibit that protons and/or free electrons could also realize the conduction in the material.

According to the reactions on the three-phase boundary, the hydrogen–metal ceramic introduces permanent modifications in the material. Such an effect could be explained by a reaction in which valence electrons are created, accompanied by the oxygen vacancies recombination. A postulated reaction involving H_2_ can be written [83,84]:

(7) $$H_{2}+2O^{2-}+V_{O}^{n-}\to {2(\mathrm{OH})}^{-}+ne^{-}.$$

(8) $$H_{2}+O^{2-}\to H_{2}O+2e^{-}.$$

(9) $$H_{2}+O_{o}^{x}\to H_{2}O+V_{O}^{••}+2e^{-}.$$

In the case of reaction (8), the response of the material would be reversible, while in the case of reaction (9), new vacancies are created, which would manifest itself in an increase in conductivity under the influence of subsequent exposure to an oxidizing atmosphere. The improved conductivity in hydrogen can be explained by the risen concentration of valence electrons and subsequent decreased conductivity in the 5th series arising from the recombination of oxygen vacancies according to Equation (7).

Another possible explanation of GDC behavior at temperatures 550–600 °C is the occurrence of reaction (9) followed by a hydration process:

(10) $$H_{2}O+V_{0}^{••}\to O_{0}^{x}+2H_{i}^{•}.$$

A coupling effect between a proton, oxygen ion, and oxygen vacancy leads to a synergistic transport effect: the migration of O^−2^ in the bulk of GDC particles in an opposite direction to promote proton migration on the GDC particle surface [85]. The change in the conduction mechanism is evidenced by a sharp rise in conductivity manifested by a change in the impedance spectrum shape visible in the admittance plane in Figure 6c above 550 °C in anH_2_-containing atmosphere.

In the literature, sensors are described in which, during exposure to ammonia gas, the response is a reversible increase in conductivity caused by an increase in the concentration of valence electrons in the material in accordance with the reaction [86,87]

(11) $${\mathrm{NH}}_{3}+2O_{O}^{2-}\to 0.5N_{2}O+1.5H_{2}O+4e^{-}.$$

The conductivity in both the 3rd and 5th series for the ScCeSZ specimen decreases, which can be explained by taking into account that the material is the oxygen ionic conductor. The comparison of spectra in both reduced atmospheres indicates that reactions run much more effectively with H_2_ than with NH_3_. The succeeding drop of conductivity in the following 4th and 5th series in the synthetic air can be elucidated by reactions resulting in oxygen vacancies recombination:

(12) $${2\mathrm{NH}}_{3}+3V_{0}^{••}+6O_{O}^{x}\to {6(OH)}_{O}^{•}+N_{2},$$

(13) $$H_{2}+V_{0}^{••}+2O_{O}^{x}\to 2{\left(OH\right)}_{O}^{•}$$

The presented results indicate that the investigated materials’ electrical properties are consistent with Arrhenius law in the synthetic air, in NH_3_ for GDC, and in H_2_ for ScCeSZ. In the atmosphere containing H_2_ for GDC and ScYbSZ, the shift in the plot is denoted. The result can be explained by a rapid change in the effectiveness of the reactions (7) and (12) above the temperatures 400 °C and 550 °C for ScYbSZ and GDC, respectively.

## 5. Conclusions

From the commercial zirconia- and ceria-based powders dedicated as solid electrolytes for SOFC, the dense sintered bodies were prepared. The materials were examined by SEM, XRD, and EDS techniques. Material based on ceria is characterized by a different microstructure than zirconia-based ceramics. In the first case, the dense, flat surface without characteristic grains was revealed, while the zirconia ceramics shows typical polycrystalline structure comprised of round, partly separated grains. In samples based on zirconia, some disagreements between the declared and experimental elemental composition were observed.

According to EIS studies conducted in oxidizing and reducing atmospheres, the zirconia samples are oxygen ionic conductors, whereas mixed-ionic electronic conduction is shown in ceria. Thus, GDC would be the most appropriate to be used as an anode element in SOFC devices working at temperatures above 500 °C. In such conditions, the effectiveness of hydrogen oxidation significantly increases. Moreover, GDC exhibits significant and reversible electrochemical response to NH_3_ in the whole investigated temperature range. Therefore, it may be prospective as material for working elements of ammonia gas sensors.

The utmost electrochemical response was recorded in the case of ScCeSZ material for exposition on NH_3_ above 400 °C. However, the reaction was only partially reversible, which limits its usability as an ammonia sensor element without further modification.

On the other hand, ScYbSZ shows the highest stability of electrical properties in reducing atmospheres below 400 °C, as well as a noticeable reversible response for H_2_ above 400 °C. On this account, it may be an interesting candidate for the working phase of an H_2_ sensor or solid electrolyte for IT-SOFC devices.

In summary, the presented studies are crucial for the evaluation of the potential usage of investigated materials as well as for determining their optimal working conditions, i.e., temperature and atmosphere composition. Nevertheless, further research on preparation and ceramic composition effects on the observed electromechanical properties is required.

## Figures and Tables

**Table 1:** Specific conductivity values of Ce(Re)O_2−_*_δ_* materials according to the literature [75].

Composition	*σ*/Ω^−1^ m^−1^ 500 °C	*σ*/Ω^−1^ m^−1^ 600 °C	*σ*/Ω^−1^ m^−1^ 700 °C
Ce_0.9_Gd_0.1_O_2−_*_δ_*	9.5 × 10^−1^	2.53 × 10^0^	5.44 × 10^1^
Ce_0.9_Sm_0.1_O_2−_*_δ_*	3.3 × 100	9.0 × 10^−1^	2.00 × 10^0^
Ce_0.9_Y_0.1_O_2−_*_δ_*	8.7 × 10^−1^	3.44 × 10^0^	1.015 × 10^1^
Ce_0.8_Gd_0.2_O_2−_*_δ_*	5.3 × 10^−1^	1.8 × 10^0^	4.700 × 10^0^

**Table 2:** Experimental phase compositions with crystallographic data obtained from XRD analysis collated with elemental composition from EDAX EDS analysis compared with theoretical values.

Specimen Label	Assumed Composition	Composition by XRD with Lattice Constant	Element	Experimental Content/%wt	Theoretical Content/%wt
GDC	Ce_0.8_Gd_0.2_O_1.9_	CeO_2_ 100% *a* = 0.54547 nm	O	12.94	17.49
			Ce	67.38	64.42
			Gd	19.67	18.09
ScCeSZ	Sc_0.1_Ce_0.01_Zr_0.89_O_1.95_	c-ZrO_2_ 96.3% *a* = 0.51254 nm CeO_2_ 3.7%	O	13.6	26.38
			Y	1.51	0.00
			Zr	72.04	68.64
			Sc	9.69	3.80
			Ce	3.16	1.18
ScYbSZ	Sc_0.09_Yb_0.01_Zr_0.9_O_1.95_	c-ZrO_2_ 100% *a* = 0.50906 nm	O	17.05	26.20
			Zr	57.90	68.95
			Sc	2.83	3.40
			Yb	22.21	1.45

**Table 3:** Increase in specific conductivities obtained in 1 series in air with risen temperatures from EIS spectra analysis and geometry of the samples.

Temperature/°C/°C	Specimen Conduction/Ω^−1^ m^−1^		
	GDC	ScCeSZ	ScYbSZ
200	2.55 × 10^−6^	4.57 × 10^−7^	3.48 × 10^−7^
300	1.51 × 10^−4^	7.44 × 10^−5^	6.42 × 10^−5^
400	2.04 × 10^−3^	4.67 × 10^−3^	2.07 × 10^−3^
450	5.61 × 10^−3^	1.91 × 10^−2^	6.88 × 10^−3^
500	1.38 × 10^−2^	5.64 × 10^−2^	1.97 × 10^−2^
550	3.01 × 10^−2^	1.37 × 10^−1^	4.74 × 10^−2^
600	7.32 × 10^−2^	2.90 × 10^−1^	1.04 × 10^−1^

## Data Availability

The original contributions presented in the study are included in the article, further inquiries can be directed to the corresponding authors.

## Abbreviations

**List of Abbreviations (In Order of Appearance)**	
YSZ	yttria-stabilized zirconia
CGO10	Ce_0.9_Gd_0.1_O_2−δ_
CGO20	Ce_0.8_Gd_0.2_O_2−δ_
GDC	Ce_0.8_Gd_0.2_O_1.9_
ScCeSZ	Sc_0.1_Ce_0.01_Zr_0.89_O_1.91_
ScYbSZ	Sc_0.09_Yb_0.01_Zr_0.9_O_1.95_
XRD	X-ray diffraction spectroscopy
EDS	energy-dispersive X-ray spectroscopy
EIS	electrochemical impedance spectroscopy
SOFC	solid oxide fuel cell
**List of Symbols (In Order of Appearance)**	
Zr^4+^	zirconium cation
*MeO*	metal in the second oxidation state oxide
${\mathrm{Me}}_{\mathrm{Zr}}^{\prime \prime }$	zirconia in the fourth oxidation state ion sitting on metal in the second oxidation state lattice site with double positive charge
$V_{0}^{••}$	double positively charged oxygen vacancy
$O_{0}^{x}$	an oxygen ion sitting on lattice site with neutral charge
${\mathrm{Me}}_{2}O_{3}$	metal in the third oxidation state oxide
Re2O3	a rare earth oxide
Ce^4+^	ceria cation in the fourth oxidation state
Ce^3+^	ceria cation in the third oxidation state
*δ*	oxygen deficiency in metal-doped oxide ceramic
*X*	Vegard’s Slope
*r_i_*	difference between the ionic radii of the dopant metal and Ce^4+^ for the coordination number equal to 8
*z_i_*	charge difference between the introduced ion and Ce^4+^ ions
*σ*	specific conductivity
2*Θ*	angle between incident beam and the crystallographic reflection plane in XRD
*A*	lattice constant
R1, R2, R3	resistor elements
CPE1, CPE2, CPE3	constant phase elements
L1	inductance element
*R*1, *R*2, *R*3	resistance parameters of resistors
*CPE1-T*, *CPE2-T*, *CPE3-T*	time parameter in constant phase elements
*CPE1-P*, *CPE2-T*, *CPE3-T*	phase parameter in constant phase element
*L*	inductance element parameter
*Z*′	real impedance component
*Z*″	imaginary impedance component
*Y*′	real admittance component
*Y*″	imaginary admittance component
*T*	absolute temperature
*t*	temperature in degrees Celsius
*q*	valency of mobile ions
*c*	relative concentration of mobile ions to the number of possible positions in the lattice
*μ*	mobility of the ions
$e^{-}$	electron
$V_{0}^{n-}$	n-time negatively charged oxygen vacancy
O^2−^	oxygen anion
*A*	a constant in Arrhenius law
*E_A_*	the activation energy for conduction
k	Boltzman’s constant
$P_{O}$	oxygen partial pressure
*m*	a parameter determined by both the type of the carrier (n or p) and the defects (e.g., oxygen vacancy) in the semiconductor
${\left(\mathrm{OH}\right)}^{-}$	hydroxide ion
*n*	numbers of electrons
$H_{i}^{•}$	proton in interstitial site
${\left(\mathrm{OH}\right)}_{O}^{•}$	hydroxide ion sitting on oxygen lattice site with positive charge
**List of Chemical Formulas (In Order of Appearance)**	
Ce_0.8_Gd_0.2_O_1.9_	assumed composition of commercial material (specimen GDC)
Sc_0.1_Ce_0.01_Zr_0.89_O_1.95_	assumed composition of commercial material (specimen ScCeSZ)
Sc_0.09_Yb_0.01_Zr_0.9_O_1.95_	assumed composition of commercial material (specimen ScYbSZ)
Ce(Re)O_2−_*_δ_*	rare earth doped ceria
NH_3_	gaseous ammonia
H_2_	gaseous hydrogen
ZrO_2_	zirconia
CO	carbon monoxide
SiO_2_	silica
MnO_2_	manganese oxide
Al_2_O_3_	aluminum oxide
CaO	calcium oxide
MgO	magnesium oxide
Y_2_O_3_	ytrria
Sc_2_O_3_	scandia
Yb_2_O_3_	ytterbium oxide
Si_3_N_4_	a silicon nitride
AlN	aluminum nitride
CuO	copper (II) oxide
c-ZrO_2_	cubic zirconia
N_2_O	nitrous oxide
H_2_O	water
